# A Review of Biomarkers Used for Assessing Human Exposure to Metals from E-Waste

**DOI:** 10.3390/ijerph16101802

**Published:** 2019-05-21

**Authors:** Aubrey L. Arain, Richard L. Neitzel

**Affiliations:** Department of Environmental Health Sciences, University of Michigan, Ann Arbor, MI 48109, USA; aubreyll@umich.edu

**Keywords:** metals, e-waste, biomarker

## Abstract

Electronic waste recycling presents workers and communities with a potential for exposures to dangerous chemicals, including metals. This review examines studies that report on blood, hair, and urine biomarkers of communities and workers exposed to metals from e-waste. Our results from the evaluation of 19 publications found that there are consistently elevated levels of lead found in occupationally and non-occupationally exposed populations, in both the formal and the informal e-waste recycling sectors. Various other metals were found to be elevated in different exposure groups assessed using various types of biomarkers, but with less consistency than found in lead. Antimony and cadmium generally showed higher concentrations in exposed groups compared to reference group(s). Mercury and arsenic did not show a trend among exposure groups due to the dietary and environmental considerations. Observed variations in trends amongst exposure groups within studies using multiple biomarkers highlights the need to carefully select appropriate biomarkers. Our study concludes that there is a need for more rigorous research that moves past cross-sectional study designs, involves more thoughtful and methodical selection of biomarkers, and a systematic reporting standard for exposure studies to ensure that results can be compared across studies.

## 1. Introduction

### 1.1. Electronic Waste (E-Waste) Hazards

Electronic waste (“e-waste”) refers to discarded electronic products, including personal electronics and appliances [1]. E-waste is the fastest growing waste stream globally, with an estimated 44.7 million metric tons produced globally in 2016, worth approximately $65 billion US dollars [2,3]. Informal e-waste recycling is an emerging economy mainly in low- and middle-income countries, as much of the e-waste produced in higher-income countries is recycled through formal methods, or is exported to other countries [4,5,6].

Electronic waste is recycled to recover raw materials and resalable parts that can be sold to create income. In addition to valuable materials such as gold and silver, e-waste contains many types of hazardous chemicals that are released during the recycling process [6,7]. While many of these chemicals are harmful to human and ecological health, this review will focus specifically on metals. Electronics contain metals that are toxic in any dose, such as Pb, Cd, and Hg, as well as essential trace metals like Fe, Zn, Mn, and Cu, which are only toxic in doses higher than is required for normal metabolic functions [8]. Electronic waste also includes metalloids, some of which are also essential trace elements in the human body. For the purposes of this review, we will refer to metals, metalloids, transition metals, and post-transition metals as simply “metals”.

### 1.2. Assessing Exposures to Metals in E-Waste

Humans can be exposed to metals from e-waste through multiple routes, including ingestion, inhalation, and dermal absorption. Elevated levels of metals have been found in the environment [5,9,10,11,12,13,14,15,16] and agricultural products near e-waste sites [17,18,19]. The tendency of workers to engage in e-waste recycling in informal settings, often near areas where they live or eat, suggests a variety of potential exposure pathways to metals from e-waste recycling [20,21]. Ingestion of contaminated environmental media and agricultural products presents one exposure pathway. Surface dust contamination and suspended metal particulates from e-waste recycling have been found in work sites as well as non-work sites, presenting a potential inhalation, ingestion, or dermal exposure [22,23,24,25].

The heterogeneity of composition of electronics and different ways in which people come into contact with e-waste makes characterization of e-waste exposures challenging. Summaries of the different metals found in e-waste products can be found in the literature (for example, see [6,26]); these represent a source of information about the potential for human exposures, but additional assessment is required to understand actual exposures. The use of biomarkers, such as concentrations of metals in hair, urine, or blood, enables researchers to understand the relative doses of different metals for different e-waste exposure groups. However, when selecting a biomarker to quantify metal levels in humans, it is important to consider the exposure routes, metabolic pathways, and appropriate biological sample type to correctly assess exposures.

### 1.3. Objective of Review

The objective of this review was to examine the current literature and compare the concentrations of various metal biomarkers in exposed e-waste populations. We sought to evaluate the differences in exposures between occupational and non-occupational exposure settings, and which metals present the greatest human exposure risk in the e-waste context. We made comparisons between metal types, concentrations, and biomarker selections in different studies. We included a study evaluation tool and discussion of the state of the literature to evaluate knowledge gaps for future studies.

## 2. Materials and Methods

### 2.1. Data Source and Search Strategy

**Figure 1:**
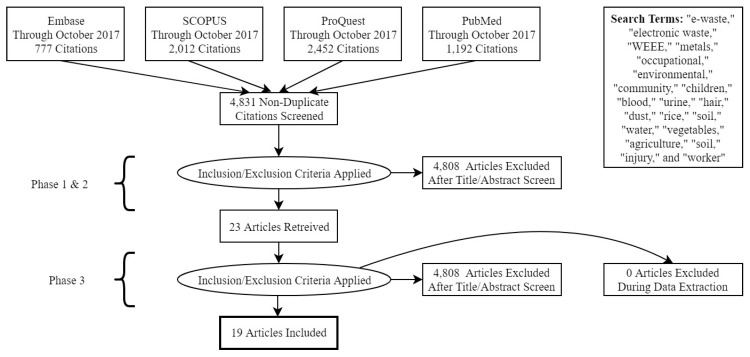
Flow diagram illustrating database search and study selection methodology.

An overview of our methodology can be found in Figure 1. English-language articles published through October 2017 were retrieved after searching four databases: Embase, SCOPUS, ProQuest, and PubMed. One study published in 2018 was included as it was published online ahead of print. Broad search terms were used to capture all studies that included adult human biomarker samples (Figure 1). The first three terms along with “metals” were searched in the title and abstract, while the Boolean term “and” was used to search for any of the last fifteen terms in the full text.

### 2.2. Study Selection

Study selection occurred in 3 phases. In phase 1, titles of all papers were screened for relevance. Titles were excluded if they did not focus on metal exposures, did not include an adult human biomarker, were reviews, were not published in a peer-reviewed journal, or were not relevant. At the conclusion of the title screen, 23 studies advanced to phase 2, the abstract screen. Abstracts were further screened for indications that human biomarkers were measured, and quantitative data presented. Finally, in phase 3, the remaining 21 full texts were further evaluated for the inclusion of an adult human metal biomarker in an e-waste-exposed population, leading to a total of 19 articles included in the review.

### 2.3. Data Extraction

Data extracted from studies included study design, recruitment information, demographic data, analysis methods, summary statistics from biomarkers, main results, and conclusions of biomarkers. The most pertinent data are summarized in Table 2.

### 2.4. Study Quality Assessment

To assess the quality of studies with regard to the purposes of this review, the authors modified a version of the Newcastle–Ottawa Scale (NOS) for cross-sectional studies developed by Elyasi et al. [27]. The tool was adjusted to evaluate the validity of analytical methods (instead of outcome measure validity) and to include a reference or comparison group. Furthermore, the tool was used only to evaluate metal biomarkers within studies, and thus does not reflect any additional non-biomarker measurements, such as environmental samples. The NOS tool and explanation of scoring can be found in Supplementary Table S1.

## 3. Results

### 3.1. Biomarkers and Metals

The 19 included studies included five types of biomarkers (whole blood, serum, plasma, hair, and urine) and 32 different metals (Table 1). Plasma (*n* = 2) and serum (*n* = 4) were the least common biomarkers, and nine of the 19 studies examined more than one category of biomarker. Lead (*n* = 14) and cadmium (*n* = 12) were the most commonly studied elements.

Table 2 presents basic details and overall conclusions for each study. Studies ranged in year of publication from 2009 to 2018, representing both formal and informal e-waste recycling in 6 different countries, with China and Ghana being best represented (*n* = 8 and 6 studies, respectively). Study populations ranged from 19–448 participants, with 3–205 participants per exposure group. Tang et al. [40], reported a total study population of 67 participants but did not report sample sizes for individual groups. At least 4 studies incorporated children, but because they were child workers grouped together with adult workers, the studies were included. The tables below report groups by exposure type, including occupationally exposed (workers in e-waste), non-occupationally exposed (those who are exposed to e-waste through activities not including e-waste work), reference groups (those who may be exposed to one or more types of metal but not through e-waste activities), and control groups (those who are not believed to be exposed to any significant source of metals). Only 5 studies reported smoking rates for the exposure groups. All but two studies were cross-sectional [34,43].

### 3.2. Modified Newcastle-Ottawa Scale

Study scores ranged from 2–9 out of a possible score of 11 (Table 3). Three studies reported using random sampling techniques, two studies reported non-random sampling techniques, and the remaining fourteen studies either used convenience sampling or did not provide a description of their methods. Amankwaa et al. [28] was the only study to justify their sample size. No study reported comparability and response rate between participants and non-participants. All but one study, Li et al. [35], reported incorporating quality control and validation techniques in their protocol. With the exception of one study, Ceballos et al. [30], all studies used at least one exposure comparison group, and four studies included at least two comparison groups. Six studies did not control for confounders, while three studies only controlled for only a single confounder. All but two studies were found to have appropriate statistical tests.

### 3.3. Data Extraction Results

Additional elements not found in the sections below can be found in Supplementary Table S1.

#### 3.3.1. Lead

Fourteen studies measured Pb (Table 4) using all five biomarkers relevant to this review (i.e., whole blood, blood plasma, blood serum, urine, and hair). The highest whole blood lead level (BLL) was found in Noguchi et al. [37], in the occupational and non-occupational exposure groups of a lead acid battery recycling community in Vietnam, with the maximum value of both sampling events being more than ten times the reference level of 10 μg/dL. This same population also had the highest median BLL values. Dartey et al. [31] had a similar max and median BLL value for their sample of lead acid battery workers. The non-occupational male control group in Dartey et al. [31] had a slightly higher median BLL than the e-waste recycling workers exposure group. Both studies that examined formal e-waste recycling in high-income countries reported maximum BLLs above the 10 μg/dL reference level [30,34,48].

One study reported Pb concentrations in serum (Table 4). A suitable reference value was not found for Pb serum, but any level of lead in blood is undesirable. The four exposure groups retained their relative position from largest to smallest values for geometric mean (GM) in urine and serum biomarkers, but not whole blood [31]. Plasma Pb levels showed a higher concentration in the non-occupationally exposed study group compared to the control group. Both groups had a mean Pb plasma concentration profoundly higher than the reference value of <0.1 μg/dL in a non-exposed population from Sweden.

All measures of central tendency for urinary lead were above the reference value of 0.5 μg/g creatinine (Table 4). However, only one study corrected for specific gravity, and two of the studies did not adjust for creatinine or specific gravity [29,34,39]. Similar to the whole blood results, the formal e-waste recycling population from Sweden had significantly elevated urinary Pb levels compared to the reference level and the reference exposure group of office workers [34].

Informal and formal e-waste recyclers (GM = 9.07 and 16.1 μg/g, respectively) from India had hair Pb concentrations above the 6.3 μg/g reference level corresponding with a German population study [32] (Table 4). In addition to the highest BLL, the worker population from Vietnam also had the highest hair Pb concentration at 2300 μg/g, and a median hair concentration of 51 μg/g compared to 1.9 μg/g in the reference population. Tokumaru et al. [41] (2017) found a significantly higher concentration of hair Pb in the non-occupationally exposed group (mean = 66 μg/g) than the control group (mean = 2.24 μg/g).

#### 3.3.2. Cadmium

Twelve studies measured Cd in biomarkers (Table 5). Of the six that measured blood Cd concentrations, only one reported any values above the 5 μg/L reference level, which was the maximum value in a formal e-waste recycling population in the USA, at 17 μg/L [30]. Urinary Cd levels were mostly below the urinary reference level of 5 μg/g. Zhang et al. [45] reported maximum urinary Cd levels in exceedance of the reference value for both mothers of male and female newborns of mothers without occupational exposure (maximum = 16.47 and 14.39 μg/g, respectively); however, the mean values are well below the reference level for these groups (mean = 1.38 and 1.59 μg/g, respectively). Ha et al. [32] found higher levels of hair Cd in the formal e-waste worker exposure group than the informal group (GM = 0.44 and 0.05 μg/g, respectively). The formal e-waste group exceeded the reference range of 0.004–0.17 μg/g, proposed in a study of a healthy Canadian population. Tokumaru [41] reported a mean hair Cd level of 0.32 μg/g in the non-occupational exposure group, substantially higher than the mean value of 0.06 μg/g reported in the control group. Finally, Zheng et al. [46] found higher levels in the occupational group than the non-occupational exposure group, which in turn had higher levels of hair Cd than the control group (GM = 1.15, 0.34, and 0.05 μg/g, respectively).

#### 3.3.3. Antimony, Arsenic, Mercury

Results for antimony, arsenic, and Hg biomarker concentrations can be found in Table 6. These elements are grouped together as they are all non-essential elements in humans. In five of the six studies reporting Sb concentrations in blood, urine, and hair biomarkers, the occupationally- and non-occupationally exposed groups consistently had higher concentrations of Sb than the reference and control groups, with occupational exposure groups reporting the highest values. Julander et al. [34] reported higher urinary median Sb levels in the occupationally exposed group (0.18 and 0.26 μg/L) compared to the non-occupationally exposed group (0.12 and 0.09 μg/L), but blood Sb levels were similar in both groups. Huang et al. [33] reported hair Sb concentrations that were hundreds to thousands of times larger (439.53, 389.66, and 87.96 μg/g for the occupational, non-occupational, and control groups, respectively) than the values in the other two studies, despite reporting in the same units of measurement. Additionally, the hair Sb levels in Huang et al. [33] were much larger than the proposed reference range for hair Sb of 0.003 to 0.13 μg/g.

Mercuryconcentration was examined in eight studies. Schecter et al. [38] (2017) found significantly higher levels of blood Hg in the control group, which the authors attributed to differences in diet between the two groups. Julander et al. [34] (2014) found higher levels of urinary Hg in the exposed population compared to the reference group, while the other five studies that examined urinary Hg found no significant differences between exposure groups. Two of the four studies that examined hair Hg found higher levels in the e-waste exposure groups compared to the control groups [32,36], but all hair mercury levels were below the reference level of 2.2 μg/g. Two studies examined methyl-mercury levels in addition to total mercury levels [38,40].

#### 3.3.4. Copper, Iron, Nickel, and Zinc

Results for Cu, Fe, Ni, and Zn, four metals essential to human health, are displayed in Table 7. Ten studies examined copper levels. In both the blood and serum biomarkers there was a large magnitude of difference in the values reported between studies, as well as with the reference value of 100–150 μg/L serum [58]. Dartey et al. [31] reported similar values between blood and serum Cu levels. Wang et al. [62] reported serum Cu levels similar to those of Dartey et al. [31], while Julander et al. [34] reported plasma Cu levels that were within the same range as the serum Cu levels reported by Srigboh et al. [39]. Reported urinary Cu values were near the reference value of 4.3–12.2 μg/g [53], with the exception of Asante et al. [29] whose reported values ranged from 77–278 μg/g. Tokumaru et al. [41] reported a significantly higher mean level of Cu in hair of the occupational group compared to the reference population (75 μg/g and 12.3 μg/g, respectively). Ha et al. [32] and Zheng et al. [46] also found higher levels of Cu in the hair of occupational and non-occupational reference groups compared to the control group.

Seven studies examined Fe concentrations. Wang et al. [42] found significantly elevated blood Fe levels in serum of both male and female occupational exposure groups compared to male and female reference groups (Asante et al. [29] found significantly higher concentrations of Fe in urine in the occupational exposure group compared to the reference groups (130, 44, and 57 μg/L, respectively), while Dartey et al. [31] found no significant difference between groups. Finally, Tokumaru et al. [41] found significantly higher concentrations of Fe in the hair of the occupational exposure group compared to the reference group (95.5 and 43.5 μg/g, respectively).

Six studies examined Ni in biomarkers. Wittsiepe et al. [44] was the only study to find a significant difference between exposure groups with regards to Ni. The urinary Ni concentration of the occupational exposure group was higher than the reference group (3.18, 2.03 μg/g, respectively).

Eight studies examined Zn in biomarkers. There was a large discrepancy in the values reported. Dartey et al. [31] reported values approximately one–one thousandth of the whole blood reference value [59]. Srigboh et al. [39] reported whole blood values that were lower, but were within the same level of magnitude, as the reference level. Julander et al. [34] and Li et al. [35] found higher levels of zinc in the reference population. A similar trend was found in urinary Zn, with several studies reporting a higher concentration in the referent populations compared to the control populations [29,34,39]. Julander et al. [34] found a slightly elevated urinary Zn concentration in e-waste workers after the first sampling event, and a slightly higher concentration in the referent population after the second sampling event. All three studies examining hair biomarkers of Zn found e-waste workers to have the highest concentrations, but all were within the proposed reference range [32,41,46,53].

#### 3.3.5. Other Metals

Additional elements beyond those described above were reported in some studies (Table 1). Results for cobalt, chromium, Mn, and molybdenum are displayed in Supplementary Materials Table S2. Although multiple studies reported on these four metals, no clear exposure-related trend was found. Other trace elements of note include indium, which Dartey et al. [31] found to be elevated in the urine samples of both occupational exposure groups. Bismuth concentration in hair was elevated in both formal and informal e-waste workers in the study by Ha et al. [32]; silver was elevated in only the informal worker hair samples. Tokumaru et al. [41] found higher levels of vanadium and tin in the hair of e-waste workers, and higher levels of Sr and Ba in the hair of the control group.

## 4. Discussion

### 4.1. Trends in E-Waste Exposure amongst Groups

There is consistent evidence in the 19 reviewed studies of human exposure to unsafe levels of metals resulting from occupational, and, to a lesser-extent, non-occupational activities. Several studies showed occupational exposures to certain metals to be higher than non-occupational exposures, which in turn are still elevated compared to control or other referent groups. However, some studies found levels of different elements were higher in the non-occupationally exposed or control groups.

Formal and informal recycling practices both appear to present dangerous levels of exposures to worker health. Ceballos et al. [30] found elevated BLL in formal recycling industry workers sampled in the USA. Similarly, Julander et al. [34] found elevated blood and urinary Pb, blood Cr, blood Co, and urinary Hg in workers compared to non-workers. Ha et al. [32] found elevated levels of hair Cu, Sb, and bs in both informal and formal recycling groups when compared with control groups; hair Hg and Ag were elevated in the informal group, and hair Pb and Mo in the formal group. It is frequently asserted that formalizing the e-waste sector will reduce worker exposures [62,63,64]. However, our results suggest that the current practices and controls in the formal sector are still not sufficiently protective.

Several studies attempted to assess differences in occupational exposures by task. Dartey et al. [31] suggested that elevated Cd and Sn concentrations in the e-waste workers occupational group may be due to soldering work during repair of electronics. Huang et al. [33] found that workers engaged in circuit baking had the highest mean concentration of hair Sb, followed by workers with the task of cutting plastic, selecting e-waste, and splitting e-waste. Ni et al. [36] found that workers who engaged in circuit baking had the highest median hair Hg concentration, with workers who select e-waste, split e-waste, and cut e-waste plastic having similar concentrations. Amankwaa et al. [28] found that the job with the highest mean BLLs was e-waste burner, followed by middlemen, dismantlers, collectors, scrap dealers, and repairers. Srigboh et al. [39] found difference between 16 different biomarkers in four different job tasks, with e-waste burners most commonly having the highest concentrations. In the formal industry, Julander et al. [34] found differences in air concentrations of Cd, Cu, In, and Mo depending on work task. Finally, Ceballos et al. [30] also found differences in Pb and Cd concentrations in personal air samples dependent on the type of task being performed in the formal industry. Additional stratification of biomarkers and exposure pathway sampling (air, dust, dermal, etc.) based on task will further elucidate specific hazards of e-waste recycling.

### 4.2. Metals in Populations Exposed to E-Waste

Leadwas elevated in the occupational and/or non-occupational exposure groups, but not the reference population, in at least one type of biomarker for all 14 studies in which it was measured. Blood lead levels of children living in or near e-waste sites have been reported at unsafe levels [65,66]. Given its damaging health impacts on current and future generations, its environmental persistence, and the evidence of exposure shown in this review, Pb would appear to be the metal of primary concern in e-waste recycling [67,68,69].

The results of our study did not suggest that there was chronic exposure to Cd by e-waste workers and communities. Urinary Cd is a biomarker of long-term exposure to Cd. With the exception of Zheng et al. [45], the concentrations of urinary Cd were all below the 5 μg/g BEI reference level [54]. Of the 12 studies that looked at Cd biomarkers, five found statistically significant concentrations of Cd in the exposed groups compared to the reference groups, while one study found significantly higher levels of Cd in the reference group (See Table 1 and Table 4). Given the poor reporting of other important factors in Cd exposure, e.g., smoking, more evidence is needed to determine how large of an issue Cd exposure is amongst occupationally and non-occupationally exposed populations.

Four of the five studies that examined Sb biomarkers found higher levels in the exposed groups compared to the reference groups (See Table 6). Huang et al. [33] reported a concentration of hair Sb that was thousands of times larger than the proposed reference value and larger than results found by the other two studies that reported on hair Sb levels [32,41,53]. The reason for this apparent discrepancy is unclear. Antimonyis ubiquitous in the environment, used heavily in electronic products, and is classified as a priority pollutant by the Environmental Protection Agency [70,71]. Antimony exposures appear to be associated with e-waste activities and the limited evidence found in this review suggests that further investigations in e-waste populations are warranted.

Inorganic As exposure can result from drinking water or occupational exposures, while organic arsenic exposures are commonly associated with diet. The consideration of inorganic As and its metabolites, MMA and DMA, is crucial to accurately assessing occupational As exposures. However, of the five studies that examined urinary As, only two examined the different species of As present. Results from Schecter et al. [32] agree with results from Asante et al. [39] showing that DMA and AB were the largest contributors to total arsenic. These results suggest that the main contributors to As exposure in this population were dietary.

Similar to As, Hg exposure often occurs from dietary exposure, and therefore it is important to differentiate between MeHg and THg to reduce confounding [72]. Two studies speciated mercury in their samples. Schecter et al. [38] (2017) found higher levels of THg and MeHg in the control group, which the authors concluded reflected differences in diet. Tang et al. [40] found higher levels of IHg in the hair of industrial-scale e-waste workers, and higher levels of MeHg in the two reference groups, indicating that the Hg exposure of the workers was likely occupational while the Hg exposure of the reference groups was dietary. In both cases, the measurement of Hg species resulted in more highly resolved conclusions on the differences between exposure groups.

Several of the trace elements shown in Table 1 were only measured in one or two studies. Future e-waste studies should consider incorporating fewer common metals such as silver, aluminum, beryllium, and platinum to help scientists understand which trace elements should be monitored in these populations.

### 4.3. Recommendations for Future Research

There is substantial room for improvement in the current literature on e-waste exposures, and on exposure assessments in general. First, more rigorous study design, improved sampling methods, power calculations to justify sample size, and comparisons between participants and non-participants would improve the generalizability of study results. Most of the studies we identified used a cross-sectional design (See Table 2). The limitation of cross-sectional studies to establish causal relationships is exacerbated in many e-waste settings, where there are potentially high background levels of metals in non-exposed populations due to other low-income job types, polluted environments, poor diet, poor access to health care, low socioeconomic status, poor regulations, etc. [5,6,7,73]. Studies should report their methods of recruitment and participation rates.

Second, the use of survey instruments and statistical techniques to examine confounders and effect mediators would improve the ability of researchers to evaluate links between exposure to chemicals and elevated levels in biomarkers. For example, less than half of the studies reported smoking rates for the different exposure groups, and sex composition between exposure groups was often different (See Table 2). Both factors are important in exposure to, and metabolism of, different metals, and without controlling for these factors it is difficult to determine what portion of the difference between exposure groups is due to the e-waste.

Third, a standardized set of guidelines for reporting summary statistics in biomarkers would facilitate the comparison of results across studies. Among the studies included here, unit conversions were sometimes required to enable comparison. In some cases, a conversion was not readily available. Similarly, it was sometimes not possible to compare results due to the differences in reported statistics. If journals mandated or encouraged reporting of a basic set of statistics, comparison across studies would be more easily facilitated. At a minimum, we recommend the required reporting of the geometric mean, geometric standard deviation, and the 95th percentile for all biomarker measurements collected in e-waste exposure studies.

Lastly, it is difficult to interpret the concentrations of biomarkers as there is a lack of standard reference ranges for healthy adults. Complicating this issue further, variables such as individual metabolism, nature of the biomarker (e.g., excretion rate), and timing of exposure (e.g., constant versus intermittent) can make it difficult to interpret and rely on cross-sectional biomarker measures. Consequently, researchers are often forced to select a reference range from the literature in what might not be a suitable reference population. While common, well-researched elements such as Pb or Cd have reference values for most biomarker types, it can be extremely difficult to find similar statistics for less studied elements and biomarkers. This is problematic as the interpretation of exposure results often depends upon the reference values. Similarly, selection of biomarkers should be carefully be considered, as some, such as hair lead, are not considered acceptable in environmental health.

## 5. Conclusions

The results of this review provide a summary for the current state of knowledge regarding adult exposures to metals through e-waste activities. Electronic waste presents a real and growing human health threat across the globe, including within the formal sector. It was established here that lead is a serious concern in e-waste populations. Cadmiumand Sb showed an overall trend of higher exposures in the exposed group compared to reference group(s). There is a need for more rigorous study design and implementation and reporting of published analytical methods. A field-wide standardization of reporting summary statistics, justification of biomarker selection, and universal reference values would improve comparability across studies. Future exposure studies should focus on trace metals, job tasks, and effect biomarkers.

## Figures and Tables

**Table 1:** Summary of biomarker measurement types for the study set including total values. Gray cells indicate the presence of biomarker or element in each study.

Publication	Biomarkers					Element																															
	Whole Blood	Serum	Plasma	Urine	Hair	Ag	Al	Sb	As	Ba	Be	Bi	Cd	Cr	Cs	Co	Cu	Fe	I	In	Ga	Hg	Pb	Mg	Mn	Mo	Ni	Pt	Rb	Se	Sn	Sr	Tl	U	V	W	Zn
Amankwaa et al., 2017 [28]																																					
Asante et al., 2012 [29]																																					
Ceballos et al., 2017 [30]																																					
Dartey et al., 2017 [31]																																					
Ha et al., 2009 [32]																																					
Huang et al., 2014 [33]																																					
Julander et al., 2014 [34]																																					
Li et al., 2014 [35]																																					
Ni et al., 2014 [36]																																					
Noguchi et al., 2014 [37]																																					
Schecter et al., 2017 [38]																																					
Srigboh et al., 2016 [39]																																					
Tang et al., 2015 [40]																																					
Tokumaru et al., 2017 [41]																																					
Wang et al., 2010 [42]																																					
Wang et al., 2011 [43]																																					
Wittsiepe et al., 2017 [44]																																					
Zhang et al., 2018 [45]																																					
Zheng et al., 2011 [46]																																					
**Total**	9	4	2	10	8	2	1	5	6	2	1	4	12	7	2	6	10	7	1	4	3	10	14	2	6	5	6	1	2	5	4	2	4	1	5	2	8

**Table 2:** Summary results of the study set, including exposure groups, demographics, results, main conclusions, and conclusions from sample types other than metal biomarkers.

Publication	Country	Industry	Exposure Group	Total *n*	Male *n* (%)	Child *n* (%)	Age Mean (SD)	Age Range	Smokers *n* (%)	Elevated Metals	Main Conclusion(s)
Amankwaa et al., 2017 [28]	Ghana	Informal	Occupational	81	NR	NR	NR	NR	NR	B–Pb	Workers who burned e-waste had highest BLLs. No significant difference between exposure groups, indicating environmental exposures are important. Years in e-waste, weekly work hours, residence, and frequency of changing work clothes significantly correlated with BLL.
			Non-occupational	33	NR	NR	NR	NR	NR	B–Pb	
			Control group	14	NR	NR	NR	NR	NR		
Asante et al., 2012 [29]	Ghana	Informal	Occupational	20	20 (100)	NR	27	15–42	5 (25)	U–Fe, Sb, Pb	Concentrations of Fe, Sb, and Pb in urine of e-waste workers significantly higher than reference levels after interaction by age, indicating that workers are exposed through recycling. Primary species in urine were arsenobetaine and dimethylarsinic acid and both were positively correlated with total arsenic and with each other. Relative concentration of As in urine was high but was low in water, suggesting common exposure source for As compounds, probably fish and shellfish.
			Reference group—gold mining	25	3 (12)	NR	45	19–81	1 (4)		
			Non-occupational	3	2 (67)	NR	30	16–40	0 (0)		
Ceballos et al., 2017 [30]	USA	Formal	Occupational	46	NR	0	NR	NR	NR	B–Pb	Pb and Cd were primary metals of concern, but this may differ in time and place given variability of e-waste recycling stream, where additional metals can be present.
Dartey et al., 2017 [31]	Ghana	Informal	Occupational—LBRW	64	64 (100)	0	31.8	20–49	2 (3)	S–Pb; B–Pb; U–Pb, I, Sb;	B-Co, Se, and Hg elevated in whole population. Concentrations of B-Hg highly associated with B-Se and As, indicating fish consumption as a common source. Higher concentrations of Cd and Sn may be related to soldering during repair work, while higher S-Mn, Cr, and Ni may point to welding. U-Cr, Hg, and Sn negatively associated with BMI.
			Occupational—ERW	64	64 (100)	0	32.6	18–50	0	S–Mn, Cr; U–As, I, Sn	
			Non-occupational—Male referent group	65	65 (100)	0	30.2	18–50	1 (2)		
			Non-occupational—FPT	26	0	0	34.2	20–49	0	B–Cu; S–Cu, Se; U–Co	
Ha et al., 2009 [32]	India	Both	Occupational—Informal	5	5	0	NR	NR	NR	H–Cu, Sb, Bi, Hg, Ag	Elevated levels from recycling sites compared to control sites suggest exposure to those elements found at both sites (Cu, Sb, Bi) may be common to recycling activities, whereas differences between sites (Mo, Pb, Hg, Ag) suggest site-specific exposures that might be caused by differences in methods of recycling used.
			Occupational—Formal	6	6	0	NR	NR	NR	H–Cu, Sb, Bi, Pb, Mo	
			Control group	8	8	0	NR	NR	NR		
Huang et al., 2014 [33]	China	Informal	Occupational	138	197 (96) ^1^	0	23.07 (7.9) ^1^	NR	NR	Sb	Sb concentrations highest in e-waste workers, then non-occupationally exposed group, compared to control group.
			Non-occupational	67	197 (96) ^1^	0	23.07 (7.9) ^1^	NR	NR	Sb	
			Control group	80	55 (68.8)	0	21.90 (0.8)	NR	NR		
Julander et al., 2014 [34]	Sweden	Formal	Occupational—BL	53	46 (87)	0	NR	NR	NR	B–Pb, Cr; U–Pb, Hg;	Few differences found in exposure patterns between different work tasks. Rare metals must be monitored as well as more well-known metals. Correlation between some metals (Sb, V, Hg, In, Pb) in air samples and biomarkers.
			Non-occupational—BL	10	8 (80)	0	NR	NR	NR		
			Occupational—FU	25	18 (72)	0	NR	NR	NR	B–Pb, Co; U–Pb, Hg;	
			Non-occupational—FU	7	5 (71)	0	NR	NR	NR		
Li et al., 2014 [35]	China	Informal	Non-occupational	30	16 (53)	NR	41 (11.0)	NR	0	B–Cu, Pb, Mg	Non-occupational exposure group had reduced beneficial minerals (Ca, Zn) and increased Pb, Cu, and Mg compared to the control group. Pb levels in the non-occupational exposure group were 50% higher than control group, indicating chronic Pb poisoning.
			Control group	28	14 (50)	NR	33 (2.1)	NR	0		
Ni et al., 2014 [36]	China	NR	Occupational and Non-occupational	205	197 (96)	0	23.07 (7.9)	NR	NR	H–Hg	Male participants significantly more likely to have higher Hg concentrations in both exposure groups. Living near e-waste activities for a long time and working with e-waste may be important contributors to Hg in hair.
			Control group	80	55 (68)	0	21.9 (0.8)	NR	NR		
Noguchi et al., 2013 [37]	Vietnam	Informal	Occupational and Non-occupational—Sampling event 1	49	16 (33)	0	NR	NR	NR	B–Pb; U–Pb; H-Pb;	Males had higher levels of Pb in blood, urine, and hair; likely from task differences. Participants from all exposure groups had BLLs above 10 μg/dL and were comparable to other areas known to be contaminated with dangerous levels of Pb.
			Occupational and Non-occupational—Sampling event 2	93	30 (32.3)	23 (24.7)	NR	NR	NR	B–Pb;	
			Control group—Urban—Sampling event 1	20	9 (45)	0	NR	NR	NR		
			Control group—Rural—Sampling event 1	71	24 (33.8)	5 (7)	NR	NR	NR		
Schecter et al., 2017 [38]	Vietnam	Informal	Occupational	40	0	0	median 39	18–52	0	B–Pb; U–Pb;	B–Pb, Cd and Hg in both exposure groups were elevated compared with NHANES. Higher levels of Hg and MeHg among the control group likely due to differences in diet. Occupational exposure to Pb occurred among recyclers. Exposure to As, Pb, and Hg was environmental.
			Control group	20	0	0	median 37	18–52	0	B–Hg, B–MeHg;	
Srigboh et al., 2016 [39]	Ghana	Informal	Occupational	58	58	0	25.9 (7.9)	NR	NR	B–Cd, Pb; U–As	Many blood and urinary elements were within reference ranges. B–Cd, Pb, and U–As were elevated compared to background populations elsewhere. Workers who burned e-waste had highest biomarker levels.
			Non-occupational	11	0	0	26 (12.8)	NR	NR		
Tang et al., 2015 [40]	China	NR	Occupational—Industrial scale e-waste	NR	NR	NR	NR	NR	NR	H–IHg	High MeHg in control group and industrial-scale e-waste group likely due to heavier fish consumption. Higher T–Hg and I–Hg in small-scale group likely from work exposures. Highest mean concentrations of T–Hg and I–Hg in acid bath workers, followed by workers who burn electronics, dismantlers, and administrators.
			Non-occupational—small-scale e-waste	NR	NR	NR	NR	NR	NR		
			Non-occupational—Industrial scale e-waste	NR	NR	NR	NR	NR	NR	H–MeHg	
			Control group	NR	NR	NR	NR	NR	NR	H–MeHg	
Tokumaru et al., 2017 [41]	Ghana	Informal	Non-occupational	56	54 (96)	NR	32	6–65	NR	H–V, Fe, Cu, Mo, Cd, Sn, Sb, Pb	Isotopic ratios indicate that Pb originated from contaminated soils, fish, and foodstuff. Humans living around e-waste site more exposed to certain metals (see column to left), and these elements were included in same cluster during analysis; they could have originated from contaminated soil at e-waste site.
			Control group	10	7 (70)	NR	20	13–33	NR	H–Mg, Sr, Ba	
Wang et al., 2010 [42]	China	Informal	Occupational—Females	100	0	0	47.01 (0.6) ^3^	NR	(26) ^3^	B–Fe	Both occupational exposure groups had significantly increased B–Fe levels compared to control group, but not compared to non-occupationally exposed group. Drinking was significantly correlated with elevated lg B–Fe.
			Non-occupational—Females	54	0	0	51.28 (1.5) ^3^	NR	(10) ^3^	B–Fe	
			Control—Females	59	0	0	54.85 (0.6) ^3^	NR	NR		
			Occupational—Males	98	98 (100)	0	47.01 (0.6) ^3^	NR	(26) ^3^		
			Non-occupational—Males	34	34 (100)	0	51.28 (1.520) ^3^	NR	(10) ^3^		
			Control—Males	32	32 (100)	0	54.85 (0.6) ^3^	NR	NR		
Wang et al., 2011 [43]	China	Informal	Occupational and Non-occupational	48	34 (71)	0	37.2 (8.1)	NR	25 (52)	B–Pb	Length of time spent working with e-waste or living near an e-waste site may contribute to an increase in BLLs.
			Control	56	31 (55)	0	39.6 (8.2)	NR	25 (45)	U–Cd	
Wittsiepe et al., 2017 [44]	Ghana	Informal	Occupational	73	61 (84)	NR	26.1 (9.6)	NR	(22) ^2^	B–Pb; U–Ni, Cd, Cr.	BLLs were elevated in both exposure groups, and the occupational exposure group had significantly higher BLLs than the control group. Exposure to Hg, Pb, Cr, and Ni in Ghana is higher than German background levels.
			Control group	37	29 (78)	NR	25.2 (7.4)	NR	(10) ^2^		
Zhang et al., 2018 [45]	China	NR	Non-occupational—mother/male newborn	123	0	NR	26.29 (4.27) ^4^	NR	1 (0.4) ^4^	U–Cd	Maternal U–Cd levels in non-occupational exposure groups higher than other populations, both inside and outside of China, indicating that the elevated U–Cd levels were the result of environmental Cd contamination in the e-waste site of Guiyu, China.
			Non-occupational—mother/female newborn	113	0	NR	26.29 (4.27) ^4^	NR	1 (0.4)	U–Cd	
			Control group—mother/male newborn	111	0	NR	28.52 (4.33) ^4^	NR	2 (1) ^4^		
			Control group—mother/female newborn	101	0	NR	28.52 (4.33) ^4^	NR	2 (1) ^4^		
Zheng et al., 2011 [46]	China	Informal	Occupational	40	NR	0	NR	NR	NR	H–Cd, Cu, Pb	Order of concentrations of metals in hair was: Zn > Pb, Cu > Cd > Ni, with highest levels found in the occupational exposure group. Elevated Cd, Pb, and Cu levels among occupational group likely to have originated from e-waste recycling activities. The distribution pattern of heavy metals in hair samples revealed that children are particularly vulnerable to heavy metal pollution caused by e-waste.
			Non-occupational	46	37 (80)	22	NR	NR	NR	H–Cd, Cu, Pb	
			Control group	39	34 (87)	11	NR	NR	NR		

NR = Not Reported; H = Hair; B = Blood; S = Serum; U = Urine; LBRW = Lead Acid Battery Recycling Workers; ERW = Electronic Recycling Workers; FPT = Female Petty Traders; BL = Baseline; FU = Follow-Up; BLL = Blood Lead Levels; PBDE = Polybrominated Diphenyl Ether; PCB = Polychlorinated Biphenyls; NHANES = National Health And Nutrition Examination Survey; THg = Total Mercury; MeHg = Methyl Mercury; IHg = Inorganic Mercury; ^1^ Data available only for occupational and non-occupational exposure groups combined. ^2^ From larger dataset (Feldt et al. [47] (2014)). ^3^ Male and female exposure groups pooled. ^4^ Mothers of male/female newborns pooled.

**Table 3:** Categories and results of the modified Newcastle–Ottawa Scale for cross-sectional studies.

	Sample Selection (4 Stars)			Analysis (2 Stars)	Comparability (4 Stars)		Outcome (1 Star)	Total (11 Stars)
Publication	1. Representativeness of Sample: a ** Random; b * Non-Random; c Selected Groups; d No Description	2. Sample Size: a * Justified and Satisfactory; b Not Justified	3. Non-Respondents: a * Comparability and Response Rate Satisfactory; b Comparability and/or Response Rate Unsatisfactory; c No Description	4. Exposure Measurement: a ** Validated Method; b * Non-Validated Method but Method Available or Described; c No Description	5. Comparison Group: a * Described by Authors as Geographically Distinct; b * Same Community; c No Comparison Group	6. Subjects in Outcome Groups Comparable: a * Study Controls for Most Important Confounder; b * Study Controls for Any Additional Confounder; c Study Did not Control for Any Confounder. d No Comparison Group	7. Statistical Test: a * Clearly Described and Appropriate; b Not Described, not Appropriate, or Incomplete	
Amankwaa et al., 2017 [28]	b *	a *	c	a **	a * b *	a * b *	a *	9
Asante et al., 2012 [29]	d	b	c	a **	a*	a*	a *	5
Ceballos et al., 2017 [30]	d	b	c	a **	c	d	b	2
Dartey et al., 2017 [31]	d	b	c	a **	b *	a * b *	a *	6
Ha et al., 2009 [32]	d	b	c	a **	a *	c	b	3
Huang et al., 2014 [33]	d	b	c	a **	a *	a * b*	a *	6
Julander et al., 2014 [34]	d	b	c	a **	b *	c	a *	4
Li et al., 2014 [35]	a **	b	c	b *	a *	a * b *	a *	7
Ni et al., 2014 [36]	c	b	c	a **	a *	a * b *	a *	6
Noguchi et al., 2013 [37]	d	b	c	a **	a *	a * b *	a *	6
Schecter et al., 2017 [38]	d	b	c	a **	a *	a * b *	a *	6
Srigboh et al., 2016 [39]	c	b	c	a **	b *	c	a *	4
Tang et al., 2015 [40]	d	b	c	a **	a * b *	c	a *	5
Tokumaru et al., 2017 [41]	c	b	c	a **	a *	c	a *	4
Wang et al., 2010 [42]	a **	b	c	a **	a * b *	a *	a *	8
Wang et al., 2011 [43]	b *	b	c	a **	a *	a * b *	a *	7
Wittsiepe et al., 2017 [44]	a **	b	c	a **	a *	a * b *	a *	6
Zhang et al., 2018 [45]	d	b	c	a **	a *	c	a *	4
Zheng et al., 2011 [46]	d	b	c	a **	a * b *	a *	a *	6

For an explanation of scale scoring, please see Supplementary Table S1.

**Table 4:** Results of data extraction from a study set for Pb biomarkers in blood, serum, plasma, urine, and hair.

Authors	*n*							Ref Level									Ref Level							Ref Level	
		Blood (μg/dL)						10 μg/dL ^6^			Urine (μg/g)						0.50 μg/g ^7^			Hair (μg/g)				6.3 μg/g ^8^	
		Min	Max	Mean	SD	GM	Med	25th %ile	75th %ile	90th %ile	Min	Max	Mean	SD	GM	Med	25th %ile	75th %ile	90th %ile	Min	Max	Mean	SD	GM	Med
Ha et al., 2009 [32]	5																			2.38	74.5			9.07	
	6																			3.74	31.8			16.1	
	8																			0.94	19.8			2.61	
Noguchi et al., 2013 [37] ^3^	49	5.5	110				20				0.003	0.2				0.02				2.5	2300				51
	93	14	122				34																		
	20	1.9	6.3				3.3				0.0008	0.006				0.03				0.8	5.5				1.9
	71	1	11				3.3																		
Tokumaru et al., 2017 [41]	56																			3.38	408	66	79.8		
	10																			1.35	3.48	2.24	0.71		
Zheng et al., 2011 [46]	40																							40.1	
	46																							15	
	39																							2.94	
Amankwaa et al., 2017 [28]	81	0.5	18.8	3.49	3.54																				
	33	0.3	8.2	3.54	2.5																				
	14	0	0	0	0																				
Asante et al., 2012 [28] ^2^	20										0.86	18.3	7.3	6.06	4.24										
	25										0.31	33.7	3.84	2.34	6.4										
	3										2.98	7.27	4.61	4.27	2.32										
																				Serum (μg/dL)				N/A	
Ceballos et al., 2017 [30]	46	ND	14																						
Dartey et al., 2017 [31] ^1^	64	4.5	109.9			20					<DL	46		1.8						0.01	2.8			0.12	
	64	3.6	43.7			9.7					<DL	24		1.1						0.01	0.94			0.05	
	65	3.4	47.8			10.2					<DL	6.1		0.6						0.01	0.42			0.04	
	26	1.8	37.9			5.4					<DL	2.3		0.6						0.01	0.34			0.03	
Julander et al., 2014 [34] ^1, 5^	53	0.95	23				3.2				0.19	17				1.8									
	10	0.48	2.4				1.5				0.01	1.6				0.66									
	25	0.71	24				3.3				0.03	17				2.4									
	7	0.49	2.7				1.6				0.24	1.9				0.9									
Schecter et al., 2017 [38]	40						4.82									3.22									
	20						2.93									2.31									
Srigboh et al., 2016 [39] ^1, 2^	58			7.93	5.8		6.35	4.01	9.98	14.22			9		8	7	4.9	9.7	14						
	11			3.71	2.62		3.57	0.93	6	7.83			13.6		5.3	12.7	8.4	18.7	22						
Wang et al., 2011 [43] ^4^	48						11.45	9.35	14.41							41	23	71							
	56						9.1	7.28	11.39							34	23	44							
Wittsiepe et al., 2017 [44] ^1^	72	3.1	35.1	10.19			8.85			17.9															
	40	2	10.3	4.43			4.1			5.65															
																				Plasma (μg/dL)				<0.1 μg/dL ^9^	
Li et al., 2014 [35] ^1^	30																					9.04	4		
	28																					6.84	1.61		

^1^ Blood units converted to μg/dL from μg/L; ^2^ Urine reported in (μg/L) with no adjustment for creatinine or specific gravity; ^3^ Urine units converted to μg/g from ng/g. ^4^ Urine units converted to μg/g from μg/mg. ^5^ Urine sample concentration corrected using specific gravity. ^6^ Set by US Center for Disease Control and National Institute of Occupational Safety and Health [48]. ^7^ NHANES median value [49]. ^8^ 95th %ile from the German Environmental Survey 1990–1992 [50,51]. ^9^ Non-exposed population in Sweden [50]. N/A indicates that no suitable reference or comparison value was found in the literature. Ref = Reference; Min = Minimum; Max = Maximum; SD = Standard Deviation; GM = Geometric Mean; Med = Median, 25th %ile = 25th percentile, 75th %ile = 75th percentile, 90th %ile = 90th percentile.

**Table 5:** Results of data extraction from study set for Cd biomarkers in blood, urine, and hair.

Authors	*n*							Reference Level									Reference Level						Reference Level	
		Blood (μg/L)							5 μg/L ^5^		Urine (μg/g)						5 μg/g ^5^			Hair (μg/g)		0.004–0.17 μg/g ^6^		
		Min	Max	Mean	SD	GM	Med	25th %ile	75th %ile	90th %ile	Min	Max	Mean	SD	GM	Med	25th %ile	75th %ile	90th %ile	Min	Max	Mean	SD	GM
Asante et al., 2012 [29] ^2^	20										0.02	0.77	0.43	0.17	0.37									
	25										0.01	1.62	0.51	0.4	0.35									
	3										0.12	0.25	0.17	0.07	0.16									
Ceballos et al., 2017 [30] ^1^	46	<LOD	17								<LOD	1.1												
Dartey et al., 2017 [31]	64	<LOD	0.9			0.2					LDR	LDR			LDR									
	64	0.1	0.7			0.3					LDR	LDR			LDR									
	65	<LOD	1.8			0.2					LDR	LDR			LDR									
	26	<LOD	4.2			0.3					LDR	LDR			LDR									
Ha et al., 2009 [32]	5																			0.08	3.55			0.44
	6																			0.03	0.1			0.05
	8																			0.04	0.21			0.08
Julander et al., 2014 [34] ^3^	53										0.01	2.4				0.37								
	10										0.18	0.61				0.27								
	25										0.12	1.4				0.37								
	7										0.17	0.3				0.27								
Schecter et al., 2017 [38]	40						0.59									1								
	20						0.59									0.83								
Srigboh et al., 2016 [39]	58			1.7	3		1.2	0.5	1.6	3.1														
	11			1.4	0.5		1.3	1	1.7	2.5														
Tokumaru et al., 2017 [41]	56																			0.01	2.16	0.32	0.43	
	10																			0.02	0.14	0.06	0.04	
Wang et al., 2011 [43] ^4^	48						1.29	0.76	4.06							1	1	2						
	56						1.84	0.77	4.71							2	1	4						
Wittsiepe et al., 2017 [44]	72	0.2	2.1	0.55			0.51			0.87	0.01	1	0.18			0.12			0.36					
	40	0.2	1.1	0.57			0.57			0.82	0.01	0.22	0.11			0.1			0.2					
Zhang et al., 2018 [45]	123										0.05	16.47	1.38	0.74		0.92	0.55	1.66						
	113										0.1	14.39	1.59	0.92		1	0.69	1.77						
	111										0.06	2.77	0.75	0.05		0.67	0.31	1.05						
	101										0.04	3.2	0.76	0.06		0.59	0.29	0.9						
Zheng et al., 2011 [46]	40																							1.15
	46																							0.34
	39																							0.05

^1^ Blood units converted to μg/L from μg/dL. ^2^ Urine reported in μg/L with no adjustment for creatinine or specific gravity. ^3^ Urine sample concentration corrected using specific gravity. ^4^ Urine units converted to μg/g from μg/mg. ^5^ BEI level set by NIOSH [50]. ^6^ Proposed reference range from healthy Canadian population. N/A indicates that no suitable reference or comparison value was found in the literature. Min = Minimum; Max = Maximum; SD = Standard Deviation; GM = Geometric Mean; Med = Median, 25th %ile = 25th percentile, 75th %ile = 75th percentile, 90th %ile = 90th percentile.

**Table 6:** Results of data extraction from study set for Sb, As, and Hg biomarkers in blood, serum, plasma, urine, and hair.

Element	*n*	Antimony						Arsenic						Mercury						
Biomarker		Blood (μg/L)	Urine (μg/g)			Hair (μg/g)		Blood (μg/L)	Serum (μg/L)	Urine (μg/g)			Hair (μg/g)	Blood (μg/L)		Serum (μg/L)	Urine (μg/g)		Hair (μg/g)	
Ref Values		0.4 ^3^	0.6 μg/L ^3^			0.003–0.13 ^4^		2.6–17.8 ^4^	N/A	35 μg/L ^5^			0.05 ^6^	0.5 ^7^		N/A	200 μg/L ^5^		2.2 ^8^	
Authors		Med	Mean	GM	Med	Mean	GM	GM		Mean	GM	Med	Mean	GM	Med	GM	GM	Med	Mean	GM
Asante et al., 2012 [29] ^9^	20		1.1	0.89						54.4	43.4						<LOD			
	25		0.32	0.24						85.8	76.4						<LOD			
	3		0.2	0.19						201	147						<LOD			
Dartey et al., 2017 [31]	64			0.75				3.5	2.1		75			3.6		0.7	0.22			
	64			0.16				3.6	1.9		101			3.6		0.7	0.27			
	65			0.18				3.8	2.2		82			4.3		0.8	0.26			
	26			0.16				2.5	1.9		85			3.6		0.8	0.42			
Ha et al., 2009 [32]	5						0.16													0.4
	6						0.23													0.1
	8						0.02													0.19
Huang et al., 2014 [33]	138					439.53														
	67					389.66														
	80					87.96														
Julander et al., 2014 [34] ^2^	53	2.2			0.18							13			1.4			1.4		
	10	2.2			0.12							19			1.2			0.66		
	25	2.3			0.26							18			1.3			1.1		
	7	2.3			0.09							21			1.5			0.99		
Ni et al., 2014 [36]	205																		1.12	
	80																		0.65	
Schecter et al., 2017 [38]	40											42.35			2.49			0.52		
	20											46.94			3.46			0.34		
Srigboh et al., 2016 [39] ^1^	58									77.5		38.3			0.9			0.2		
	11									117.5		92.5			1.1			0.3		
Tang et al., 2015 [40]	NR																		1.19	
	NR																		0.88	
	NR																		1.52	
	NR																		1.4	
Tokumaru et al., 2017 [41]	56					0.77							0.11							
	10					0.06							0.06							
Wittsiepe et al., 2017 [44]	72																	0.18	0.46	
	40																	0.18	0.85	

^1^ Urine reported in μg/L with no adjustment for creatinine or specific gravity. ^2^ Urine sample concentration corrected using specific gravity. ^3^ Occupationally-exposed glass workers (lowest exposure group selected) from glass industry [52]. ^4^ Proposed reference range [53]. ^5^ BEI set by ACGIH [54]. ^6^ US study control group median value [55]. ^7^ Median value for adults NHANES [56]. ^8^ FAO/WHO JECFA [57]. N/A indicates that no suitable reference or comparison value was found in the literature. Med = Median; GM = Geometric Mean.Arsenic was measured in blood, serum, urine, and hair. None of the six studies that examined As found a significant difference in biomarker As concentrations by exposure groups, and four of the six studies concluded that As exposures were due to environmental and dietary exposures [29,31,38,39]. Two studies examined various As species, including monomethylarsonic acid (MMA), dimethylarsinic acid (DMA), and arseobetaine (AB) [29,38].

**Table 7:** Results of data extraction from study set for Cu, Fe, Ni, Zn biomarkers in blood, serum, plasma, urine, and hair.

Element	*n*	Cu										Fe					Ni							Zn							
Biomarker		Blood (μg/L)		Serum (μg/L)		Plasma(μg/L)		Urine (μg/g)		Hair (μg/g)		Blood (μg/L)	Serum (μg/L)	Plasma (mM)	Urine (μg/g)	Hair (μg/g)	Blood (μg/L)		Serum (μg/L)	Urine (μg/g)		Hair (μg/g)		Blood (μg/L)	Serum (μg/L)	Plasma (μg/L)		Urine (μg/g)		Hair (μg/g)	
Ref Values		1000 ^5^		100–150 ^4^		794–2023 ^6^		4.3–12.1 ^6^		8.51–34.97 ^6^		N/A	N/A	0.04–5.31 ^6^	58.73 ^7^	16.9–29.6 ^6^	0.09–4.18 ^6^		N/A	0.59–4.06 ^6^		0.51–1.53 ^8^		7900 ^4^	N/A	551–925 ^7^		44–499 ^7^		129–209 ^5^	
Authors		Med	GM	Med	GM	Mean	Med	GM	Med	Mean	GM	Med	GM	Mean	GM	Mean	Mean	Med	GM	GM	Med	Mean	GM	GM	GM	Mean	Med	GM	Med	Mean	GM
Asante et al., 2012 [29] ^1^	20							254							130													614			
	25							77							44													713			
	3							278							57													675			
Dartey et al., 2017 [31]	64		1		1.1			13							6		<LOD		<LOD	3.1				7.1	0.9			178			
	64		1.4		1.1			13							6.6		<LOD		0.9	2.9				7.1	0.8			234			
	65		1.5		1			14							5.3		<LOD		<LOD	2.9				7.3	0.8			232			
	26		1.6		1.5			14							11		<LOD		<LOD	3.6				6.6	0.8			243			
Ha et al., 2009 [32]	5										23																				141
	6										22.8																				141
	8										7.77																				116
Julander et al., 2014 [34] ^2^	53	760					870					460,000						0.99			1.8						710		470		
	10	800					880					470,000						0.25			1.5						810		410		
	25	740					930					460,000						0.93			1.7						710		510		
	7	760					970					450,000						1.2			1.5						840		520		
Li et al., 2014 [35]	30					17.34	μM							8.43												100.66	μM				
	28					15.2	μM							8.5												127.42	μM				
Srigboh et al., 2016 [39] ^1^	58			841					22.1			409,666									12.1			4645					511		
	11			1071					65.3			391,849									26.6			4259					1116		
Tokumaru et al., 2017 [41]	56									75						95.5						1.06								171	
	10									12.3						43.5						0.40								134	
Wang et al., 2010 [43]	100				1.26								800																		
	54				0.7								870																		
	59				1.82								700																		
	98				1.92								1280																		
	34				0								910																		
	32				1.71								680																		
Wang et al., 2011 [43] ^3^	48								26																						
	56								25																						
Wittsiepe et al., 2017 [44]	72																				3.18										
	40																				2.03										
Zheng et al., 2011 [46]	40										29.81												0.74								138.95
	46										17.67												0.59								112.51
	39										9.85												0.81								122.99

^1^ Urine reported in μg/L with no adjustment for creatinine or specific gravity. ^2^ Urine sample concentration corrected using specific gravity. ^3^ Urine values converted from μg/mg to μg/g. ^4^ Healthy adult reference range [58]. ^5^ 95th percentile reference value from a healthy male Canadian population [59]. ^6^ Proposed reference range [53]. ^7^ Mean value from 24-h urine sample of healthy adults [60]. ^8^ Reference range reported from a Polish population [61]. N/A indicates that no suitable reference or comparison value was found in the literature. Med = Median; GM = Geometric Mean.

## Supplementary Materials

The following are available online at https://www.mdpi.com/1660-4601/16/10/1802/s1, Table S1: Modified Ottawa-Newcastle Scale for cross-sectional studies; Table S2: Results of data extraction from study set for Co, Cr, Mn, and Mo biomarkers in blood, serum, plasma, urine, and hair.
