# Supplementary material for: A Review of Biomarkers Used for Assessing Human Exposure to Metals from E-Waste
Source: Int J Environ Res Public Health. 2019 May 21;16(10):1802. doi: 10.3390/ijerph16101802 (PMC6572375; doi:10.3390/ijerph16101802)
Supplement: Supplementary file 1 [file ijerph-16-01802-s001.pdf]

Table S1. Modified Ottawa-Newcastle Scale for cross-sectional studies.

**Sample selection Criteria (6 stars)**																			
1) Representativeness of the sample: a) Truly representative of the average in the population** (all subjects or random sampling); b) Somewhat representative of the average in the target population* (non-random sampling); c) Selected group of subjects; d) No description of the sampling strategy																			
2) Sample size: a) Justified and satisfactory*; b) Not justified.																			
3) Non-respondents: a) Comparability between respondents and non-respondents characteristics is established, and the response rate is satisfactory*; b) The response rate is unsatisfactory, or the comparability between respondents and non-respondents is unsatisfactory; c) No description of the response rate or the characteristics of the responders and the non-responders.																			
4) Ascertainment of the exposure: a) Validated measurement tool **; b) Non-validated measurement tool but the tool is available or described*; c) No description of the measurement tool.																			
**Comparability (4 stars)**																			
1) Comparison group: a) Described by authors as separate from exposure (i.e. different community/location)*; b) Described by authors as community exposure within the same location*; c) No comparison group.																			
2) The subjects in different outcome groups are comparable, based on the study design or analysis. Confounding factors are controlled: a) The study controls for the most important factor (select one)*; b) The study controls for any additional factor *.																			

**Outcome (1 star)**																			
2) Statistical test: a) The statistical test used to analyze the data is clearly described and appropriate, and the measurement of the association is presented, including confidence intervals and the probability level (p value)*; b) The statistical test is not appropriate, not described, or incomplete.																			
**Total (11 stars)**																			

Table S2. Results of data extraction from study set for Co, Cr, Mn, and Mo biomarkers in blood, serum, plasma, urine, and hair.

Element		Chromium								Cobalt							Manganese							Molybdenum						
**Biomarker**		**Blood (µg/L)**	**Serum (µg/L)**	**Plasma (µg/L)**	**Urine (µg/g)**			**Hair (µg/g)**		**Blood (µg/L)**		**Serum (µg/L)**	**Urine (µg/g)**		**Hair (µg/g)**		**Blood (µg/L)**		**Serum (µg/L)**	**Plasma (mM)**	**Urine (µg/g)**	**Hair (µg/g)**		**Blood (µg/L)**		**Serum (µg/L)**	**Urine (µg/g)**		**Hair (µg/g)**	
**Ref values**		**0.95[a]**	**2.7[a]**	**N/A**	**1.0[4]**			**0.12[5]**		**0.38[6]**		**N/A**	**0.16-1.14[6]**		**0.004-.014[7]**		**14-16[6]**		**N/A**	**0.63-2.26[d]**	**0.11-1.32[6]**	**0.02-0.57[7]**		**1.6[6]**		**2.2[4]**	**170[6]**		**0.01-0.03**	
**Authors**	**n**	**Med**	**GM**	**Med**	**Mean**	**GM**	**Med**	**Mean**	**GM**	**GM**	**Med**	**GM**	**GM**	**Med**	**Mean**	**GM**	**GM**	**Med**	**GM**	**Mean**	**GM**	**GM**	**Mean**	**GM**	**Med**	**GM**	**GM**	**Med**	**Mean**	**GM**
Asante et al, 2012[2]	20				19	15							1.6								3.47						83.7			
	25				8.3	2.2							0.61								2.54						38.9			
	3				19	18							0.85								4.05						53			
Dartey et al., 2017	64		<LOD			0.25				1.2		0.2	0.59				9.5		0.8					1.1		1.5	96			
	64		0.7			0.23				2.6		0.2	0.61				8.1		1					1.1		1.5	87			
	65		<LOD			0.28				2.1		0.2	0.61				8		0.8					1		1.4	118			
	26		<LOD			0.34				2.1		0.5	1.5				10.3		0.8					1		1.3	75			
Ha et al., 2009	5								0.29							0.09						1.16								0.04
	6								0.4							0.05						1.86								0.07
	8								0.42							0.11						2.11								0.03
Julander et al., 2014[3]	53	1.4		0.81			0.74				0.08			0.25				11							0.97			74		
	10	1.1		0.3			0.71				0.06			0.24				8.8							0.69			44		
	25	0.85		0.31			0.24				0.07			0.21				9.4							0.49			58		
	7	0.58		0.29			0.23				0.02			0.19				9							0.47			41		
Li et al., 2014	30																			1.15										
	28																			0.23										
Srigboh et al., 2016	58				0.9		0.9							0.9				7												
	11				2.1		2.3							4.2				8.5												
Tokumaru et al., 2017	56							0.78							0.17								7.11						0.1	
	10							0.87							0.17								2.87						0.01[1]	
Wittsiepe et al., 2017	72				0.51		0.34																							
	40				0.3		0.23																							

[1] Note that $n = 1$. [2] Urine reported in (µg/L) with no adjustment for creatinine or specific gravity. [3] Urine sample concentration corrected using specific gravity. N/A indicates that no suitable reference or comparison value was found in the literature. [4] 95th percentile reference value from unexposed referent group in occupational study. [5] Median value of healthy referent population in occupational study. [6] 95th percentile reference value from a healthy Canadian population.[7]Proposed reference range.
